# Pilot randomized controlled trial testing the influence of front-of-pack sugar warning labels on food demand

**DOI:** 10.1186/s12889-019-6496-8

**Published:** 2019-02-07

**Authors:** Felicia Jia Ler Ang, Sagun Agrawal, Eric A. Finkelstein

**Affiliations:** Program in Health Services & Systems Research, Duke-NUS Medical School, 8 College Road, Singapore, 169857 Singapore

**Keywords:** Front-of-pack labeling, Nutrition labeling, Warning labels, Sugar, Online grocery store

## Abstract

**Background:**

Front-of-pack (FOP) nutrition warning labels to identify potentially harmful foods/beverages have recently been considered in Singapore. The objective of this study was to pilot test two promising FOP warning labels intended to reduce purchases of products high in sugar to determine whether a full scale trial testing one or both these labels using actual purchases is warranted.

**Methods:**

Five hundred twelve participants ≥21 years old and residing in Singapore completed all study elements online via the NUSMart Online Grocery Store study website. The study was designed as a Randomized Controlled Trial (RCT) where consumers were randomized and asked to hypothetically shop in one of three versions of an online grocery store; 1) no FOP label (control), 2) a graphical high-in-sugar label shaped like a stop sign, or 3) a text-based warning label. The proportion of labelled products purchased (primary outcome) and all secondary measures of diet quality were calculated using participants’ orders. Ordinary Least Squares (OLS) regression was used to compare purchasing behavior across the three study arms.

**Results:**

The proportion of high-in-sugar products selected (i.e., those targeted for labelling) was largest in the no label control arm at 20%. The proportion was a non-statistically significant 2 percentage points lower (*P* = 0.146) for the high-in-sugar stop-sign label arm and 4 percentage points lower (*P* < 0.05) in the warning label with deterrent text arm. We could not reject the hypothesis of equal effectiveness of the two warning labels (*P* = 0.231).

**Conclusions:**

Results suggest that the two health warning labels have potential to reduce demand for high-in-sugar products in Singapore. Future studies should test the influence of these labels using actual purchases in efforts to identify whether either labelling strategy should be considered for adoption in the local setting.

**Trial registration:**

The American Economic Association’s registry for randomized controlled trials; AEARCTR-0003800. Registered 18 January 2019.

**Electronic supplementary material:**

The online version of this article (10.1186/s12889-019-6496-8) contains supplementary material, which is available to authorized users.

## Background

The incidence of obesity, diabetes and other non-communicable diseases (NCDs) has risen rapidly in recent years. As diet is a key risk factor for the onset of NCDs [1–4], policy-makers have been looking to identify strategies aimed at discouraging unhealthy food consumption [5–7]. Most pre-packaged foods and beverages include a nutrition information panel (NIP) to help consumers make healthier purchases. However, the NIP is difficult to decipher for many shoppers and there is scant evidence that the panel has helped to curtail rising rates of obesity and NCDs [8–10].

Singapore, the country of focus for this effort, recognized the limitations of the NIP and supplemented it with a simple front-of-pack (FOP) label termed the Health Choice Symbol (HCS). The HCS symbol was first introduced in 2001 and although optional, is strongly encouraged by the Singapore government. It offers the Health Promotion Board’s endorsement for products that are healthier options within a food category, including options that are *Lower in Sugar* and *Lower in Saturated Fat*. Although this particular label has not been systematically evaluated, FOP nutrition labels appear to outperform other forms of nutritional labels in improving consumers’ ability to find and use nutritional information in purchasing decisions [11–13]. Therefore, the HCS logos may improve a shopper’s ability to identify the healthier products even when the NIP is available on the back of the label [14]. However, these logos appear on only a small percentage of products (roughly 9%) and do not identify the worst offending foods and beverages when it comes to sugar or other harmful ingredients.

Research has shown that, compared with non-directive labels like the Nutrition Facts Panel, directive and semi-directive labels may improve consumers’ ability to find and understand nutritional information [14–17]. Consumers tend to prefer simple FOP labels and to appreciate interpretational aids like descriptors or color codes [11, 18]. However, there is some evidence that positive FOP labels targeting healthier foods may not be sufficient to discourage consumption of less healthy alternatives [14, 19]. As a result, health warning labels have recently been proposed as a complement or alternative to positive FOP food labels. Studies of text warnings for tobacco products showed improved consumer education, greater knowledge of health harms of tobacco use, and decreased purchases [20, 21]. One study showed reduced intention to purchase SSBs in the presence of SSB warning labels [22]. These studies suggest that warning labels identifying harmful ingredients or adverse effects of certain food products have the potential to improve diet quality. Therefore, as motivated by several social psychological theories, including loss-framing [23] and theories of risk perception [24], providing a clearly identifiable and salient message to consumers on which foods are highest in harmful ingredients could effectively signal which foods to avoid and thus further positively alter food purchasing patterns even in the presence of the NIP and HCS logos [25]. Several empirical studies showing the effectiveness of tobacco warning labels and warning labels on select foods provide additional support for testing such labels in Singapore [14, 20, 26–28].

**Fig. 1:**
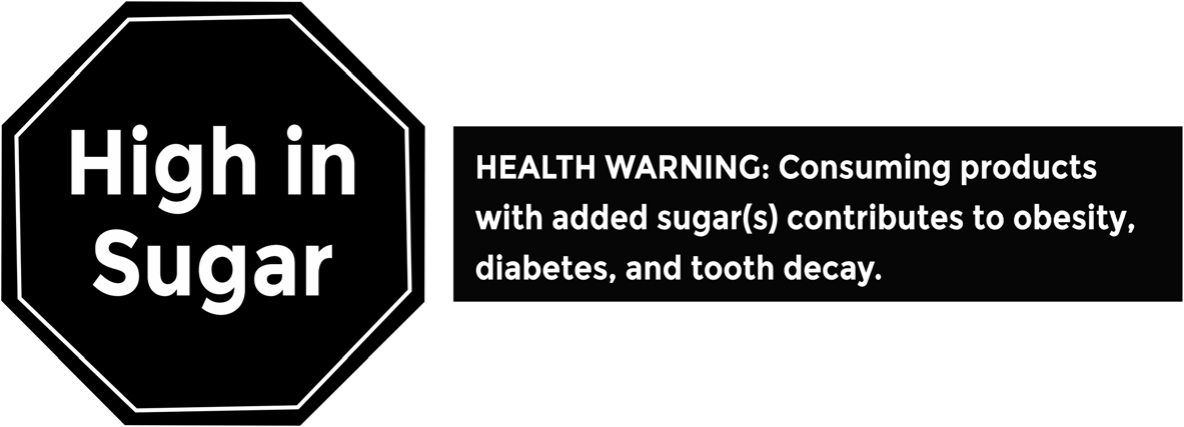
Warning labels

In this study, we use an experimental web-based grocery store to pilot test two different theory- and evidence-based ‘FOP’ warning labels aimed to reduce purchases of high-in-sugar products, even in the presence of the HCS symbol and NIP. The first label we consider is an English language version similar to the one used in Chile (Fig. 1 Left Panel) that shows a black stop sign (Arm termed SS) with the words *high-in-sugar* in the center (Fig. 1 Left Panel) [5]. The second label we consider is a text-based health warning label (Arm termed TW), which is similar to labels considered in several municipalities in the US (Fig. 1 Right Panel) and also resembles the warning label on cigarettes, but without the accompanying graphics (Fig. 1 Right Panel) [22].

Given the strongly worded language similar to tobacco warning labels that are used in the text-based health warning label, we hypothesized that the proportion of labelled (or targeted for labelling in Control Arm) products among respondents would be the largest in Control and smallest in the warning label condition. There are three reasons to assume the text based warning label will show greater effectiveness. First, it is likely to generate greater levels of loss framing through negative terminology including ‘warning’ and ‘tooth decay’. Second, because it mirrors tobacco warning labels, the negative association may lead to an implicit bias against purchasing. Finally, because of the requirement to include the full message in a readable format, it is larger than the stop sign label and therefore may be more salient to consumers. We hypothesized a similar ordering for secondary outcomes, including total sugar purchased, sugar (g) per dollar spent, and total spending.

## Methods

### Online grocery store

For this pilot and future research studies, an online grocery store (NUSMart Online Grocery Store) was developed. At the time of the pilot, NUSMart contained over 1800 non-fresh food and beverage products commonly purchased at local markets in Singapore (see Additional file 1). The web store was designed to mirror an actual web-based grocery store in look and feel. It contained products across major Food & Beverage categories, including:

- Beverages
- Dairy Products
- Cooking & Baking
- Frozen & Chilled Products
- Confectionery
- Cereals, Bakery & Spreads

All products include a picture of the item and current retail price. A subset of qualifying products also have the HCS logo displayed. The store operates similar to other on-line grocery stores, with a cart that fills as consumers shop on the store and the ability to review purchases before checkout.

**Fig. 2:**
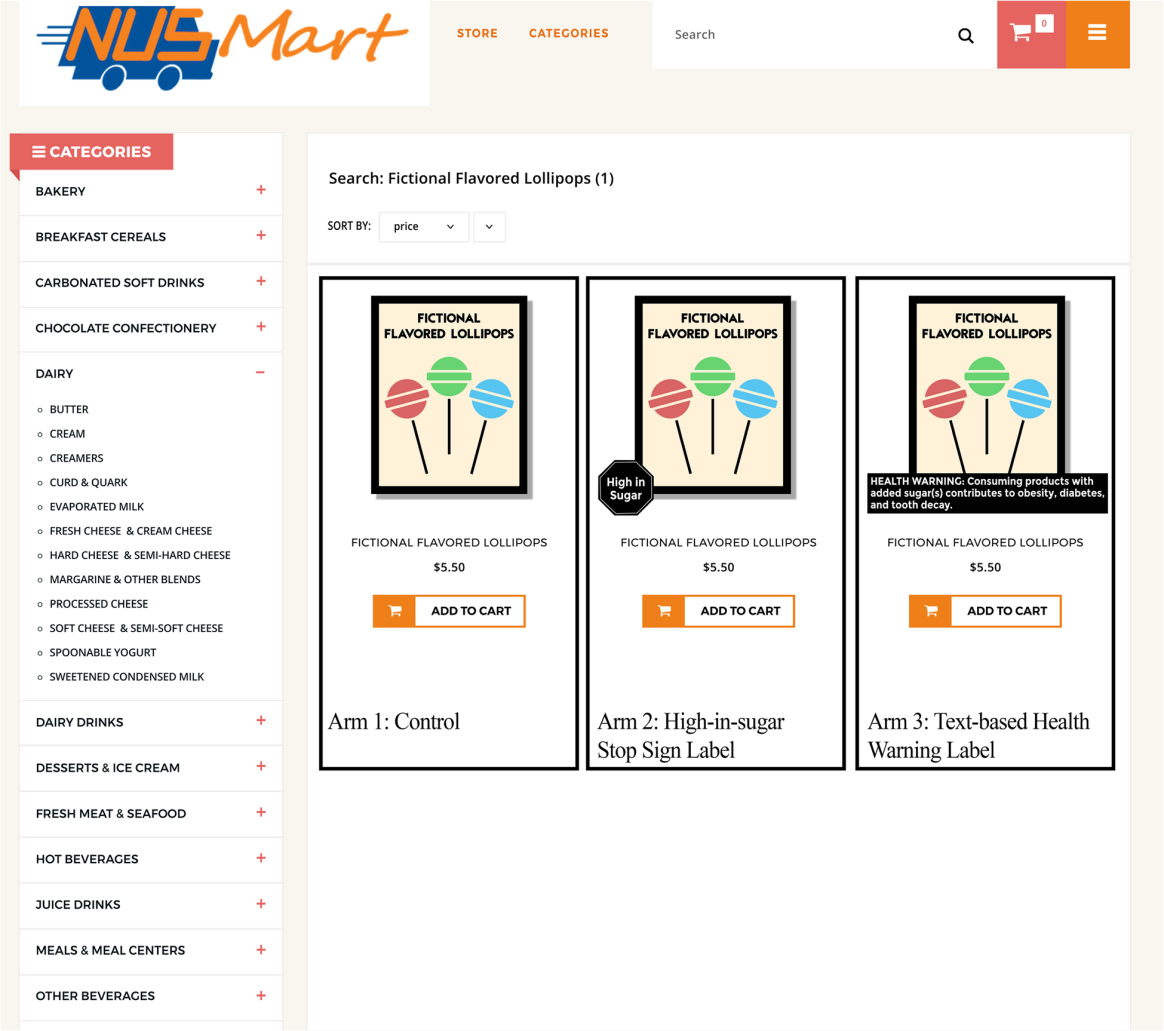
Example of the Pilot-DIET NUSMart storefront with a sample of the warning labels on the same fictional product as it appears in each study arm

We identified products to receive a warning label based on the percentage of sugar as compared to other products *within the food category*. We chose a within category approach to mirror the approach taken for the HCS program. A food product is classified as high-in-sugar if it is among the top 20% of products with the highest sugar content per 100 g within the food category. A beverage is classified as high-in-sugar if it contains at least 10 g per 100 ml (i.e. 10%) of sugar. Using this approach, 20% of food within each food category and 27% of beverages were defined as high-in-sugar and received the corresponding label for the SS and TW arms. As with the HCS symbol, the labels were displayed at the bottom of the product images (See Fig. 2). Figure 2 shows the storefront and an example of a fictional product as it appears in each arm of the study.

### Participants and procedures

We powered the study to detect differences of at least 3 percentage points across arms in terms of the percentage of high in sugar products purchased. Assuming a common group standard deviation of 0.83 (based on unpublished data from ongoing studies), an alpha of 0.05 and power of 0.8, and adjusted for multiple comparisons, a sample of 170 participants per arm was required to test our hypotheses.

**Fig. 3:**
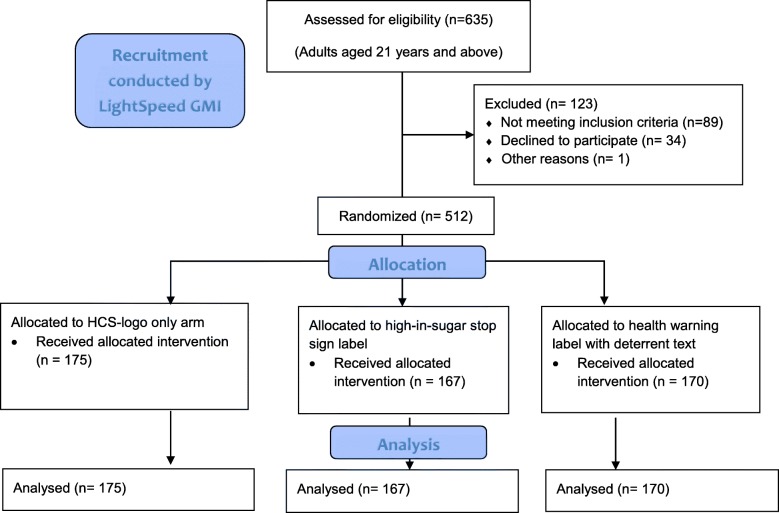
Participant flow diagram

Based on this power calculation, 512 participants were recruited anonymously from an on-line panel in August 2017. Potential participants were emailed unique links that directed them to a screener. They were excluded if they were under 21 years of age or a non-resident of Singapore. All others were offered the chance to participate. Those who consented were randomized into 1 of the 3 arms and asked to spend between S$50 (approximately 37 USD) and S$250 (approximately 183 USD) on NUSMart as if it were a real household grocery shopping trip (see Fig. 3 for diagram of study flow). A pop-up message appeared on-screen if they attempted to checkout below or above the minimum or maximum respectively. The minimum expenditure was intended to ensure sufficient purchasing data would be collected per sales order and the maximum was intended to ensure that our results were not overly influenced by a few shoppers with very large expenditures. Following the shopping task, participants completed a brief survey and were compensated according to the web panel’s in-house point system. The survey included information on age, gender, height, weight, ethnicity, presence of children in household, and whether the participant is the primary grocery shopper in the household.

### Measures

The primary outcome of interest is the proportion of high in sugar products purchased, as this is the most direct test of the influence of the labels. However, it is possible that consumers could respond to the labels by purchasing fewer labelled products but not reduce their net sugar intake, partly because a change in the labels could also influence their total spending. Therefore, we also include the following secondary outcomes:

- *Total sugar purchased (in g) per shopping trip,*
- *Sugar purchased per dollar spent (in grams per $),*
- *Total spending (in $) given that high-in-sugar products tend to be less expensive, and*
- *Total expenditure on high-in-sugar products ($).*

### Data analysis

All analyses on primary and secondary outcomes were conducted using Ordinary Least Squares (OLS) regression. A Generalized Linear Model (GLM) with logit link was also used for analysis of the primary outcome, given it represents a proportion. However, as results were identical to the OLS results, we do not report the GLM results although they are available upon request. Specifically, the outcome of interest was regressed on treatment indicators for the two labelling conditions, with the Control Arm being the omitted reference group. Results were run on the full shopping basket and separately for beverages given Sugar Sweetened Beverages are a prime target of labelling efforts [29–31]. All regressions controlled for age, gender, ethnicity, body mass index (BMI), whether the participant is a primary purchaser for the household and has children. All statistical analyses were performed using STATA.

## Results

### Data screening

Table 1 presents the characteristics of the final sample, by allocated Arm. Five hundred twelve participants were part of the final sample for the analysis. The total sample was largely Chinese (86.1%) and the mean age was 38.1 years (SD = 11.5). The average BMI was 22.8 (SD = 4.9). The majority (72.9%) reported being the primary grocery shopper for the household, and about half (46.7%) were female.

**Table 1:** Descriptive Statistics of the participants (*n* = 512) in the Pilot-DIET Study by allocated Arm

Variable	Mean/Proportion in Sample		
	Arm 1: Control (*n* = 175)	Arm 2: High-in-Sugar Stop Sign Label (*n* = 167)	Arm 3: Warning Label with Deterrent Text (*n* = 170)
	Mean (SD) or %		
Age (SD)	38.1 (11.9)	38.2 (12.2)	37.9 (10.5)
BMI (kg/m^2^) (SD)	22.4 (3.6)	24.0 (6.6)	21.9 (3.9)
Female (%)	55.4	38.9	45.3
Ethnicity (% Chinese)	85.1	86.2	87.1
Primary Purchaser (%)	72.6	75.5	70.6
Have Children (%)	42.9	46.7	44.1

Table 2 presents the regression output for each dependent variable for the full shopping basket. Both SS and TW participants purchased a lower proportion of High-in-Sugar products than Control participants but the difference was only statistically significant for TW (*P* < 0.01). Differences between SS and TW were not statistically significant (*P* = 0.231). None of the secondary outcomes (total sugar purchased (g), sugar purchased per dollar spent (g per $), total spending ($) and total expenditure on high-in-sugar products ($)) were statistically different across arms.

**Table 2:** Estimates of the Impact of Warning Labels on Measures of Diet Quality in the Pilot-DIET Study (*N* = 512)

Dependent Variable	Proportion of High-in Sugar Products (%)	Total Sugar Purchased (g)	Sugar purchased per dollar spent (g / $)	Total Dollar Spent ($)	Total expenditure on high-in-sugar products ($)
	Coefficient [Std. Error]	Coefficient [Std. Error]	Coefficient [Std. Error]	Coefficient [Std. Error]	Coefficient [Std. Error]
Constant	0.08	351.82	7.90	60.76	−4.92
	[0.04]	[179.93]	[1.77]	[13.44]	[4.82]
Stop Sign Label	−0.02	48.91	0.51	2.35	0.61
	[0.02]	[65.44]	[0.645]	[4.89]	[1.75]
Warning Label	−0.04 ***	26.18	0.36	2.01	−1.61
	[0.02]	[64.55]	[0.64]	[4.82]	[1.73]
Age (years)	0.00	10.50 ***	0.03	0.79 ***	0.27 ***
	[0.00]	[2.60]	[0.03]	[0.19]	[0.07]
BMI (kg/m^2^)	0.01 ***	10.20 *	0.10 *	−0.15	0.58 ***
	[0.00]	[5.82]	[0.06]	[0.44]	[0.16]
Female	0.02	−88.05	−1.50 ***	−2.39	2.22
	[0.01]	[56.00]	[0.55]	[4.18]	[1.50]
Chinese	−0.03 *	− 196.51 **	− 2.45 ***	− 0.60	−3.12
	[0.02]	[77.35]	[0.76]	[5.78]	[2.07]
Primary grocery Purchaser	−0.01	−47.61	0.35	−3.24	−0.68
	[0.02]	[62.21]	[0.61]	[4.65]	[1.67]
Has Children	0.01	−7.07	−0.12	1.29	−1.36
	[0.02]	[60.95]	[0.60]	[4.55]	[1.63]

* *P* < 0.1, ** *P* < 0.05, *** *P* < 0.01

Table 3 presents the regression output for each dependent variable when the sample is restricted to beverage purchases made by the 432 participants who purchased beverages. For this restricted sample, the proportion of High-in-Sugar products was 33% for the Control arm. The proportion was 6 percentage points lower (*P* = 0.085) for the SS arm and 11 percentage points lower (*P* = 0.002) for the TW arm compared to Control. As with the full sample we could not reject the hypothesis of equal effectiveness of the two warning labels (*P* = 0.172) nor were there any statistically significant differences across arms in any of the secondary outcomes.

**Table 3:** Estimates of the Impact of Warning Labels on Measures of Diet Quality for Beverage Purchases in the Pilot-DIET Study (*N* = 432)

Dependent Variable	Proportion of High-in Sugar Products (%)	Total Sugar Purchased (g)	Sugar purchased per dollar spent (g / $)	Total Dollar Spent ($)	Total expenditure on high-in-sugar products ($)
	Coefficient [Std. Error]	Coefficient [Std. Error]	Coefficient [Std. Error]	Coefficient [Std. Error]	Coefficient [Std. Error]
Constant	0.06	76.47	19.73	12.28	−3.79
	[0.10]	[132.96]	[5.43]	[7.10]	[3.52]
Stop Sign Label	−0.06 *	54.97	−0.06	0.57	0.08
	[0.03]	[44.21]	[1.81]	[2.36]	[1.17]
Warning Label	−0.11 ***	− 0.48	−2.10	1.52	− 0.25
	[0.03]	[44.22]	[1.81]	[2.36]	[1.17]
Age (years)	0.00 ***	3.06 *	−0.01	0.13	0.20 ***
	[0.00]	[1.75]	[0.07]	[0.09]	[0.05]
BMI (kg/m^2^)	0.00	4.62	−0.02	0.00	0.12
	[0.00]	[4.52]	[0.19]	[0.24]	[0.12]
Female	0.08 ***	−32.72	0.63	−4.16 **	1.07
	[0.03]	[38.29]	[1.56]	[2.05]	[1.01]
Chinese	0.04	33.14	−2.25	5.12 *	1.38
	[0.04]	[54.40]	[2.22]	[2.91]	[1.44]
Primary grocery Purchaser	−0.04	−55.38	−1.09	−0.75	−1.40
	[0.03]	[41.79]	[1.71]	[2.23]	[1.11]
Has Children	−0.02	−32.57	−0.29	−1.36	− 1.19
	[0.03]	[41.24]	[1.68]	[2.20]	[1.09]

* *P* < 0.1, ** *P* < 0.05, *** *P* < 0.01

Higher BMI is associated with a higher proportion of High-in Sugar products purchased (*P* < 0.01). Older age was associated with higher purchases of sugar (P < 0.01) and greater spending (*P* < 0.01). There were also significant effects of gender on sugar purchased per dollar spent, with women on average purchasing 1.5 g per dollar less than men (*P* < 0.01). Chinese respondents also purchased significantly less total sugar (*P* < 0.05) and sugar per dollar (*P* < 0.01) than non-Chinese.

## Discussion

The primary objective of this pilot study was to determine whether one or both of two promising FOP warning labels for high in sugar products would be worth testing further in a large-scale randomized controlled trial in Singapore using actual purchases. Results lend support to proceeding with a full scale trial as one of the two labels generated a statistically significant reduction in labelled products purchased. Our findings are consistent with recent studies examining SSB warning labels. A text-only warning label was shown to reduce purchase probabilities of SSBs, reduce perceived product attractiveness, quality and taste, and reduce perceptions of consumer “coolness” in a hypothetical experiment [32]. Another hypothetical trial focusing on warning labels for SSBs showed that the labels increase parents’ understanding of health harms associated with over-consumption of SSBs and reduce intentions to purchase SSBs for their children [22]. To date, no studies have assessed how the food labelling law in Chile has impacted purchases of sugar or other macronutrients.

Although our study only found one of the labels to be effective at influencing purchases of targeted products, a full scale trial including both labels should be pursued given that we could not reject the hypothesis that one label outperformed the other. Moreover, a full scale trial with actual purchases will allow for determining whether label that effectively influences purchasing patterns also leads to improvements in diet quality. This is relevant given that an effective label may not reduce sugar or calories purchased if consumers alter their behavior by purchasing more of unlabeled products.

As this is a pilot study, it is subject to several limitations. All shopping was hypothetical and may not generalize to actual purchases. The NUSMart store at the time this study was conducted also had fewer products than would appear in a full grocery store but more products than available in most convenience stores and it is likely that the number and type of products will influence the effectiveness of FOP labels. The NUSMart store has been expanded to include over 4000 products, which will allow for a more realistic test of the effectiveness of these labels. However, it is worth noting that even if shown to be effective (or ineffective), effectiveness may be size and venue specific and differ for web versus in store shopping. For example, the influence of labels may differ when shopping for a few products for immediate consumption, as is likely when shopping in a convenience store, compared to when making larger purchases for foods to last over a longer period of time. Effectiveness may also wane over repeated shops. Hunger and other visceral factors may also mediate the relationship between labels and food purchases regardless of venue and time to consumption. Results could also be influenced by the color, size, placement and implementation strategy (e.g., what percent of products are labelled) of the labels. In this pilot, the two warning labels were of differing sizes because we needed the health warning label to be large enough such that the text could be clearly seen on even small devices. One reason the text based label was more effective may be due to its larger dimensions. Although we could not realistically reduce the size of this label, we could test the effect of a larger stop sign label. Finally, a small sample size could have masked true but relatively small differences in outcomes between arms. Many of these concerns can be addressed by a carefully conducted 3 arm randomized trial using repeated actual, as opposed to hypothetical, purchases which is now in the planning stages.

## Conclusions

Results from the present pilot suggest that FOP warning labels have the potential to reduce demand for high in sugar products and should be tested via a full scale randomized trial. This test is warranted given that our pilot results suggest that even an effective label that reduces demand for high in sugar products may not generate a reduction in sugar or calories purchased, which is the ultimate goal of the labelling policy.

## Additional file

Additional file 1: NUSMart product categories in the Pilot-DIET study. NUSMart Category names, number of products per category, and proportions of products qualifying for the logos per category. (DOCX 14 kb)

## Abbreviations

FOP: Front-of-pack
GLM: Generalized Linear Model
HCS: Healthier choice symbol
NCDs: Non-communicable diseases
NIP: Nutrition information panel
OLS: Ordinary least ^squares^
RCT: Randomized controlled trial
SS: Black stop-sign with the words high-in-sugar
TW: Health warning label with deterring text
