# Supplementary material for: Pilot randomized controlled trial testing the influence of front-of-pack sugar warning labels on food demand
Source: BMC Public Health. 2019 Feb 7;19:164. doi: 10.1186/s12889-019-6496-8 (PMC6367807; doi:10.1186/s12889-019-6496-8)
Supplement: Supplementary file 1 — NUSMart product categories in the Pilot-DIET study. NUSMart Category names, number of products per category, and proportions of products qualifying for the logos per category. (DOCX 14 kb) [file 12889_2019_6496_MOESM1_ESM.docx]

| **NUSMart Category Names** | **Number of products per category** | **Percentage of ‘High-in-sugar’ products (%) per category** |
| --- | --- | --- |
| Hot Beverages | 166 | 38.2 |
| Dairy Drinks | 196 | 12.4 |
| Sauces & Seasonings | 287 | 20.9 |
| Meals & Meal Centers | 152 | 20.3 |
| Staples | 125 | 20.5 |
| Processed Fish, Meat & Egg Products | 111 | 21.1 |
| Pre-packaged Fruit & Vegetables | 66 | 21.2 |
| Snacks | 243 | 22.0 |
| Bakery | 267 | 20.8 |
| Spreads | 113 | 21.3 |
| Chocolate Confectionery | 119 | 22.1 |
| Dairy | 376 | 17.5 |
| Sweeteners & Sugar | 66 | 21.5 |
| Carbonated Soft Drinks | 70 | 37.3 |
| Juice Drinks | 102 | 32.0 |
| Sports & Energy Drinks | 57 | 22.6 |
| Sugar & Gum Confectionery | 114 | 18.9 |
| Soup | 68 | 21.5 |
| Breakfast Cereals | 77 | 21.6 |
| Desserts & Ice Cream | 74 | 20.3 |
| Ready-to-Drink | 74 | 10.0 |
| Other Beverages | 56 | 34.6 |
| **TOTAL** | **2979** | **21.7%** |

**Additional file 1: NUSMart product categories in the Pilot-DIET study**
